# Acute multiple toxic effects of Trifloxystrobin fungicide on *Allium cepa* L.

**DOI:** 10.1038/s41598-022-19571-0

**Published:** 2022-09-08

**Authors:** Oksal Macar, Tuğçe Kalefetoğlu Macar, Emine Yalçın, Kültiğin Çavuşoğlu

**Affiliations:** 1 Department of Food Technology, Şebinkarahisar School of Applied Sciences, Giresun University, Giresun, Turkey; 2 Department of Biology, Faculty of Science and Art, Giresun University, Giresun, Turkey

**Keywords:** Biochemistry, Genetics, Molecular biology

## Abstract

Trifloxystrobin (TFS) is a strobilurin-type fungicide that should be investigated due to its risks to non-targeted organisms. The goal of this study was to assess the susceptibility of *Allium cepa* L. to TFS in a multi-pronged approach. For 72 h, 0.2 g/L, 0.4 g/L and 0.8 g/L doses of TFS were administered to *A. cepa* bulbs and the control group was treated with tap water. The toxic effects of TFS were tested, considering physiological, cytogenetic, biochemical and anatomical analyses. TFS delayed growth by reducing the rooting ratio, root elongation and weight increase. Following TFS treatments, mitotic index (MI) scores decreased, while the formation of micronucleus (MN) and chromosomal aberrations (CAs) ascended. CAs types induced by TFS were listed according to their frequency as fragment, vagrant chromosome, sticky chromosome, uneven distribution of chromatin, bridge, nucleus with vacuoles, reverse polarization and irregular mitosis. TFS provoked an increment in superoxide dismutase (SOD) and catalase (CAT) enzyme activities as well as an accumulation of malondialdehyde (MDA). Meristematic cells of *A. cepa* roots treated with TFS had various anatomical damages, including damaged epidermis, flattened cell nucleus, damaged cortex and thickness in the cortex cell wall. All damages arising from TFS treatments exhibited dose-dependency. The findings of the present study revealed the serious toxicity of TFS in a non-targeted plant. It should not be neglected to evaluate the potential hazards of TFS with different toxicity tests.

## Introduction

Currently, the most prevalent method applied to protect plants against diseases and pests in agricultural practices is the use of pesticides^1^. New chemicals with different mechanisms of action are needed to combat the resistant pest that emerges as a result of this widespread use. One of the new fungicides developed for this purpose is strobilurins, which originate from the wood rot fungus *Strobilurus tenecellus*^2^. Owing to their ability to successfully combat a wide variety of fungal diseases, strobilurins are the best-selling agricultural fungicides worldwide^2,3^. Strobilurins, which serve in the world fungicide market with many different members and are constantly being developed, have such a broad-spectrum mechanism of action that they induce some non-targeted effects^4^.

Trifloxystrobin (TFS), as a strobilurin fungicide, binds to the region (Qo) of complex III and stops respiratory activities in mitochondria. As a result, it interrupts the vital energy cycle in fungi by inhibiting the generation of adenosine triphosphate in fungi^5^. TFS is widely used in crops such as rice, cereals, fruits, vegetables, grapes, potatoes, and soybeans to combat sheath blight and rice blast fungus^6^. Strobilurin-type fungicides such as TFS can cause environmental pollution due to their intense use, as well as their ability to remain in water, air, soil, and products after being applied to plants^7^. Despite all its advantages, TFS has a slow rate of hydrolysis in non-acidic environments, so its half-life can be several years as Trifloxystrobin acid (TFSA)^6^. In addition, TFSA, the major metabolite of TFS, is more soluble in water and is more environmentally hazardous than TFS^8^. Therefore, there is increasing concern about adverse effects on humans and non-target organisms due to TFS contamination of food and the environment. Although TFS is considered to have low toxicity to mammals, birds, and bees, there are studies showing high toxicity to aquatic and soil organisms^9^. There is a considerable knowledge gap regarding the TFS-induced toxicity on terrestrial organisms and humans.

TFS is often applied in combination with different fungicides to perform disease management on *A. cepa* L., described as the "queen of the kitchen"^10–12^. However, healthy bulbs of *A. cepa* are also an excellent test material for monitoring the damaging effects of chemicals. Due to the difficulty and ethical problems of testing the toxicity of chemicals on humans, test systems based on various types of bio-indicators have been developed. One of the most substantial indicator species is *A. cepa*, with its few and easily visible chromosomes. The administration of the *Allium* test to analyze the potential toxicity and genotoxicity of various types of chemical agents is widespread and has a long history. It is more cost-effective and provides a greater amount of data compared to animal-based assays. It also provides similar and comparable results to those obtained from animal cell lines^13^.

The aim of the present study was to monitor the toxic potency of TFS fungicide on the *A. cepa* model plant. It was achieved by evaluating the physiological, cytogenetic, and biochemical parameters. The effect of TFS on the physiology of bulbs was determined using rooting rate, root elongation, and weight gain, while chromosomal aberrations (CAs), micronucleus (MN), and mitotic index (MI) were used to evaluate the effects of the fungicide on the cytogenetic parameters of *A. cepa* roots. Levels of malondialdehyde (MDA) for lipid peroxidation and superoxide dismutase (SOD) and catalase (CAT) activities were used as biochemical indicators of toxicity. In addition, TFS-induced meristematic injuries in the root tips of *A. cepa* were also screened using cross-sections.

## Materials and methods

### Material preparation

Recently harvested *A. cepa* var. *aggregatum* bulbs (2n = 16), obtained from a local market in Giresun-Turkey. The onion bulbs used in the study were directly tested without being stored in the laboratory. Experimental solutions were prepared using a commercially available formula “Trailer” containing 50% Trifloxystrobin content (Hektaş Group, Kocaeli-Turkey). All the other chemicals used in this study were of analytical grade. The use of plants and the experiments in the present study complies with the relevant international, national and institutional guidelines.

### Experimental design

Four experimental groups were formed using healthy and similarly sized onions (11 ± 1 g) bulbs. Each group contained fifty bulbs and was exposed to aqueous TFS solutions of 0.2 g/L, 0.4 g/L and 0.8 g/L, respectively. The fungicide was applied at higher doses than in the field to monitor the possible long-term adverse effects of TFS on non-target organisms under laboratory conditions and in a short time. TFS doses were determined by preliminary studies considering the rooting ratio and root elongation. The control group was treated with tap water. Bulbs were placed in glass tubes filled with respective solutions and kept in the dark at room temperature for 72 h. Solutions were renewed daily.

### Determination of physiological effects

At the end of the experimental period, rooting ratio, root elongation and weight increase were calculated to investigate the physiological changes caused by TFS in bulbs. To calculate the rooting rate, the ratio of the number of rooted onions to the total number of onions was determined, and the result was given as a percentage^14^. The mean weight increase and root elongation of ten randomly selected bulbs from each group were measured using a precision scale and a ruler, respectively^15^.

### Determination of genotoxic effects

In order to investigate the cytotoxic and genotoxic effects caused by TFS, the changes in MN and CA frequencies were determined along with the MI value. All of these parameters were analyzed on the same slides prepared by the method suggested by Staykova et al.^16^. When the three-day rooting period was over, 1 cm of the tips of the newly emerging roots of *A. cepa* were taken and fixed with Clarke's fixator (3: ethanol/1: glacial acetic acid) for 2 h. The roots were hydrolyzed for 12 min at 60 °C in 1 N HCl in a hot water bath before being stained for 16 h with a 1% acetocarmine solution. The preparations examined under the light microscope (Irmeco, IM-450 TI) were prepared by the squash preparation method. Changes in MN and CA formations were determined by counting 100 cells in each of the ten slides (1000 cells in total) selected from each group. Changes in MI values were determined by counting 1000 cells in each of the ten slides (10,000 cells in total) selected from each group.

### Determination of biochemical effects

The malondialdehyde (MDA) level was monitored as an indicator of lipid peroxidation in cell membranes. Analyses of MDA levels in the samples were made according to the method of Unyayar et al.^17^. 0.5 g of sample material was homogenized in 1 mL of trichloroacetic acid (TCA) (5%) and then centrifuged for 15 min at 12,000 rpm at room temperature to obtain the supernatant. The mixture containing equal volumes of supernatant TCA (20%) and thiobarbituric acid (0.5%) was boiled for 25 min. After the reaction was stopped, the tube containing the mixture was placed in an ice bath and centrifuged at 10,000 rpm for 5 min. The absorbance of the newly obtained supernatant was determined at 532 nm. MDA levels were expressed as micromolar per gram of fresh weight. Lipid peroxidation analysis was carried out in triplicate.

In order to determine the oxidative balance in the cells exposed to TFS, SOD and CAT activities were monitored along with the MDA accumulation. Extraction of the enzymes was performed according to the method of Zou et al.^18^. Root materials (0.2 g) freeze-shocked with liquid nitrogen were ground in sodium phosphate buffer (2 mL; 50 mM; 7.8 pH). In order to separate the supernatant, a centrifugation process (14,000 rpm; 4 °C; 20 min) was carried out. The supernatant containing both SOD and CAT enzymes was collected. The method for analyzing the SOD activity was slightly modified from the assay of Beauchamp and Fridovich^19^. Sodium phosphate buffer (1.5 mL; 0.05 M; 7.8 pH) was mixed with a pre-mixed solution containing distilled water, EDTA-Na_2_, riboflavin, methionine, nitroblue tetrazolium chloride and polyvinylpyrrolidone. After the enzyme (0.01 mL) was added to the medium, the tube containing the reaction medium was placed in front of a fluorescent lamp with an intensity of 375 μmol/m^2^/s. After allowing the reaction to continue for 15 min, absorbance was measured at a wavelength of 560 nm. The total SOD activity was calculated and presented as unit per mg of fresh weight. Each step of the analysis was performed ten times. The method for analyzing the CAT activity was modified from the assay of Beers and Sizer^20^. Sodium phosphate buffer (1.5 mL; 0.2 M; 7.8 pH) was mixed with a pre-mixed solution containing hydrogen peroxide and distilled water. The reaction depleting hydrogen peroxide was allowed to initiate by adding the enzyme (0.2 mL) to the medium. The decrease was observed instantaneously in a spectrophotometer at a wavelength of 240 nm. The total CAT activity was calculated and presented as OD_240_ nm minute per gr fresh weight. Each step of the analysis was performed ten times.

### Determination of meristematic injuries

Pesticide residues were eliminated by washing the roots. After the cleaning step, the cross-sections of the roots were taken manually to elicit the anatomical differences between the groups. Methylene blue dye (3%) was dropped onto the cells to stain them. The preparations containing the stained root sections were examined under the light microscope (Irmeco, IM-450 TI). The intensity of the injuries induced by TFS was expressed as: undamaged, slightly damaged, moderately damaged and critically damaged.

### Statistics

Data from the experimental analyses (expressed as mean ± standard deviation in the tables) were subjected to ANOVA and Duncan’s test system (SPSS 23 Software) to determine whether there were significant differences between the means (p < 0.05).

## Results and discussion

### Physiological effects of TFS

The rooting ratio, root elongation level and weight increase of the bulbs were determined in order to find out the effects of TFS on physiological parameters (Table 1). The rooting ratio of the bulbs in the control group was 100%, indicating that extremely healthy bulbs were used in the experiment. In the treatment groups, as the TFS dose increased, the rooting ratio of the bulbs decreased. Indeed, the group treated with 0.8 g/L TFS showed only a 45% rooting ratio. Dose dependence on growth retardation was also observed in root elongation and weight increase, similar to rooting ratio. The root elongation degree of the TFS 0.2 g/L group decreased to half that of the control group, while the maximum suppression in root elongation was determined in TFS 0.8 g/L. Although the initial mean weights of the groups were close to each other, there were significant differences between the final weights. The reduction levels in the weight increase of the groups were 33% in the TFS 0.2 g/L group, 49% in the TFS 0.4 g/L group and 71% in the TFS 0.8 g/L group compared to the control group. The only factor affecting the overall weight gain was the length of the roots, as leaves still did not appear at the end of 72 h of treatment. No deformation, water loss or color change was observed in the roots or bulbs. Strobilurins, including TFS and Azoxystrobin, are marvellous guards inhibiting spore germination to protect seeds and vegetative plant parts^21,22^, when used in the proper doses and methods. However, Anstis and Wicks^23^ demonstrated that Azoxystrobin may trigger phytotoxicity depending on application method and rate in onion plants. It has also been proven that TFS can exert phytotoxic effects on some grape cultivars such as *Vitis labrusca* and *Malus* trees^24,25^. It has also been reported that the phytotoxic effects of the fungicide are exacerbated during drought stress^25^. Our results were in agreement with the study of Rao et al.^26^, which showed that the mixture of TFS and Tebuconazole caused evident growth retardation in *Capsicum annuum* L. seedlings. TFS is a chemical that has a limiting effect on mitochondrial respiration by blocking the electron transport system (ETS). Since all plants have this system as eukaryotic organisms, TFS has at least partial suppression of ETS, as previously shown in mitochondria isolated from *Triticum aestivum* L.^27,28^. Nason et al.^29^ reported that the blocking site of strobilurins in ETS is the cytochrome bc1 complex, resulting in diminished ATP production. The results of this study suggest that TFS-induced restriction in mitochondrial energy production may prevent the production of energy required for growth. The impairment in mitochondrial energy gain may have slowed down growth events such as root elongation and weight gain by inhibiting both cell division and water uptake in the plant. The increase in bulb weight is directly related to root development as it provides the necessary water for plant growth.

**Table 1:** Effect of Trifloxystrobin on selected physiological parameters.

Groups	Rooting ratio (%)	Root elongation (cm)	Weight increase (g)	Initial weight (g)	Final weight (g)
Control	100	10.6 ± 1.89^a^	+ 6.87^a^	10.88 ± 0.92–17.75 ± 1.94	
TFS 0.2 g/L	80	5.3 ± 0.67^b^	+ 4.60^b^	10.97 ± 0.72–15.57 ± 0.68	
TFS 0.4 g/L	60	3.0 ± 0.80^c^	+ 3.52^c^	10.85 ± 1.03–14.37 ± 0.71	
TFS 0.8 g/L	45	1.2 ± 0.46^d^	+ 2.00^d^	10.80 ± 1.01–12.80 ± 0.84	

The averages shown with different letters (a–d) in the same column are significantly different at p < 0.05.

### Cytogenetic effects of TFS

**Figure 1:**
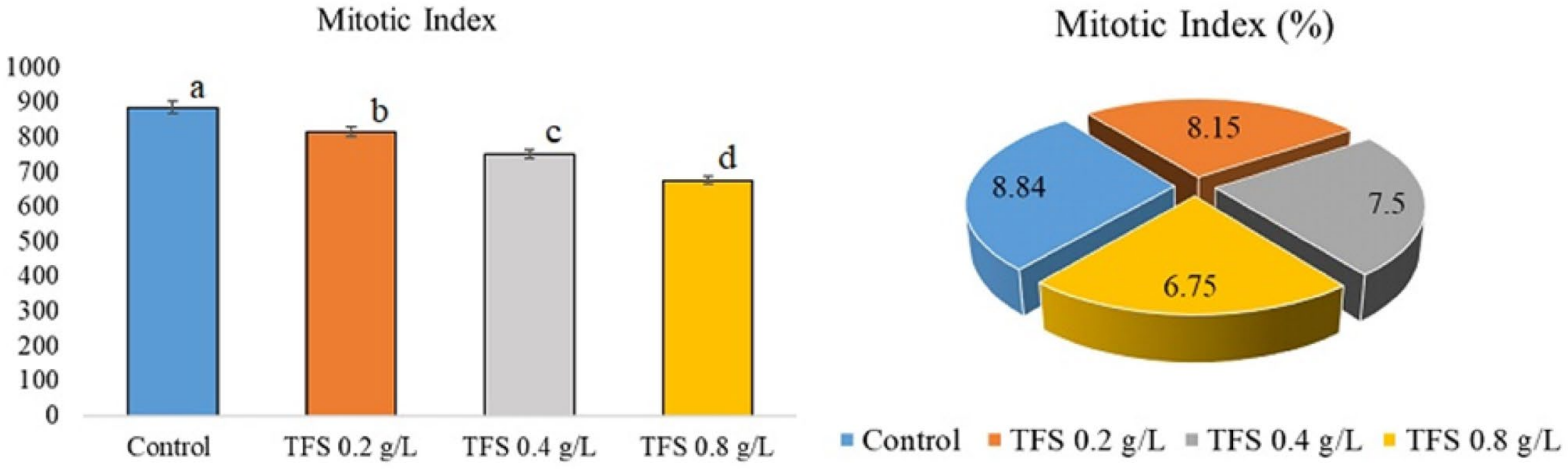
Changes in MI induced by Trifloxystrobin. The averages shown with different letters (a–d) are significantly different at p < 0.05).

Genotoxicity is one of the endpoints for manifesting the toxic potential of pollutants^30^. The results of the genotoxicity analysis showed that TFS was genotoxic from the lowest dose to the highest dose administered to *A. cepa*. MI is a parameter utilized to estimate the changes in cell proliferation rate due to the cytotoxic effect of pollutants^31^. All doses of TFS caused significant and concentration-dependent decreases in MI (Fig. 1). The slowdown in root elongation may be a direct consequence of the reduction in MI. Many researchers^32–34^ has been reported fungicide-related MI decrement in *A. cepa*, whereas the effect of TFS on MI of onion cells has not been studied so far. Yet, cytotoxicity assays demonstrated that TFS was 50 to 150-fold more cytotoxic than the triazoles in Chinese hamster ovary cells^35^. Morejohn et al.^36^ reported that pesticides inhibit microtubule synthesis and reduce the rate of mitosis by interacting with plant tubulin proteins. Therefore, it is thought that the decrease in MI may be due to TFS blocking microtubule synthesis.

**Figure 2:**
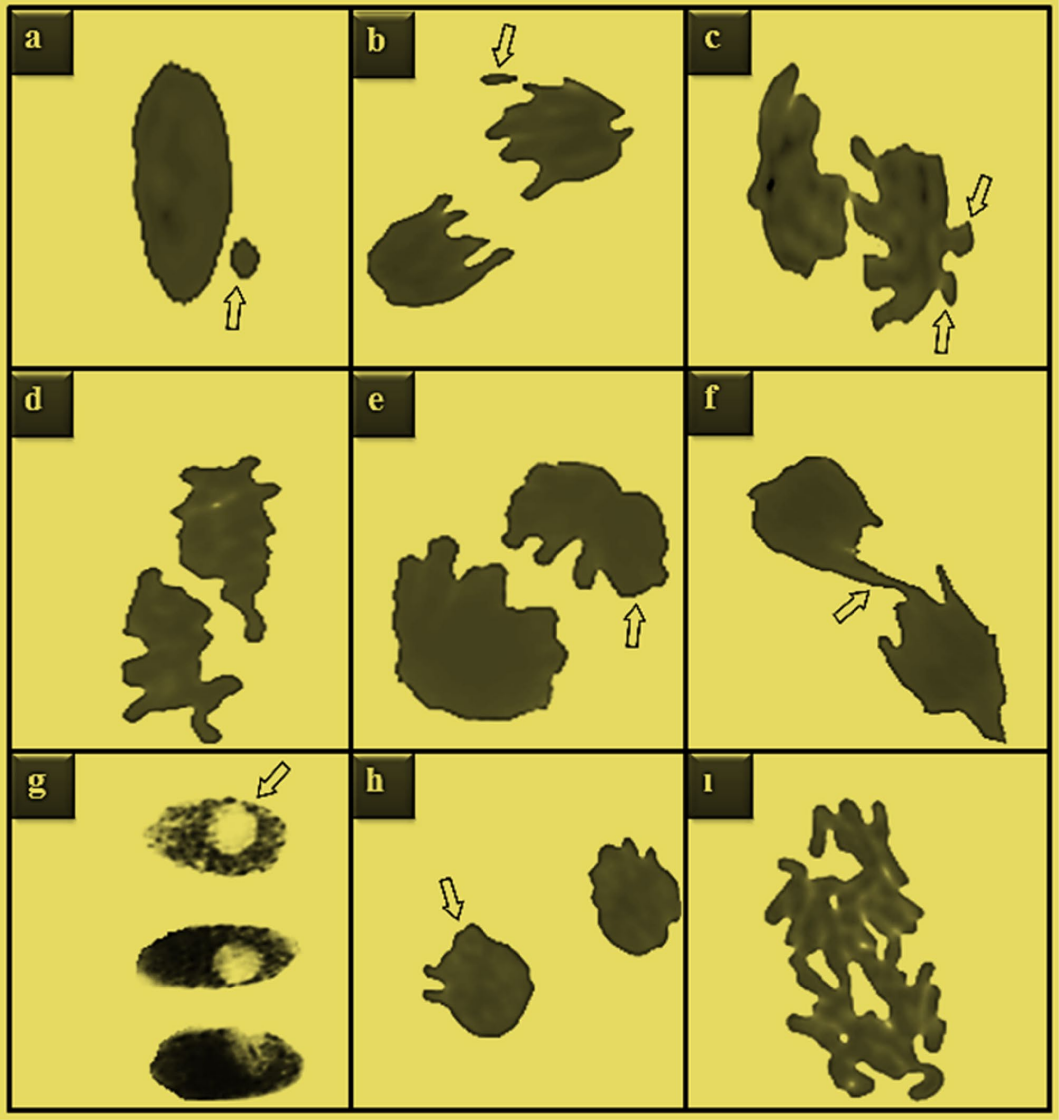
Trifloxystrobin-induced chromosomal damages. MN (**a**), fragment (**b**), vagrant chromosome (**c**), sticky chromosome (**d**), uneven distribution of chromatin (**e**), bridge (**f**), nucleus with vacuoles (**g**), reverse polarization (**h**), irregular mitosis (**i**).

The fungicide led to a significant increase in MN formation in all groups exposed to TFS (Fig. 2a, Table 2). While MN was not observed in the control group, the frequency of MN increased as the exposure dose of TFS increased in the treatment groups. The MN test is a well-established method for screening disorders at the chromosomal level and relies on identifying rounded, tiny and stainable inclusions known as “micronuclei”^37^. It is a reliable marker of cytogenetic anomalies that appear following the influence of genotoxic agents^30^. The results of our study confirmed the report by Liu et al.^5^, which suggested that TFS induces biochemical toxicity as well as genotoxicity. Similarly, Çayır et al.^38^ revealed that Pyraclostrobin, another member of the strobilurin family, accelerates the formation of MN in human lymphocytes.

**Table 2:** Genotoxicity induced by Trifoxystrobin.

Damages	Control	TFS 0.2 g/L	TFS 0.4 g/L	TFS 0.8 g/L
MN	0.00 ± 0.00^d^	18.9 ± 1.25^c^	31.3 ± 2.76^b^	55.6 ± 4.67^a^
FG	0.00 ± 0.00^d^	16.5 ± 1.18^c^	28.8 ± 2.70^b^	50.2 ± 4.58^a^
VCR	0.00 ± 0.00^d^	12.6 ± 0.92^c^	25.4 ± 2.51^b^	44.8 ± 4.12^a^
SCR	0.18 ± 0.43^d^	11.3 ± 0.85^c^	21.9 ± 2.33^b^	38.5 ± 3.89^a^
UDC	0.12 ± 0.28^d^	9.5 ± 0.77^c^	18.4 ± 1.97^b^	33.2 ± 3.54^a^
BR	0.00 ± 0.00^d^	7.3 ± 0.68^c^	15.7 ± 1.50^b^	28.4 ± 2.95^a^
NVC	0.00 ± 0.00^d^	5.8 ± 0.54^c^	12.2 ± 1.30^b^	20.3 ± 1.96^a^
RPL	0.00 ± 0.00^d^	4.5 ± 0.46^c^	10.3 ± 0.98^b^	17.6 ± 1.53^a^
IM	0.00 ± 0.00^d^	3.0 ± 0.38^c^	7.1 ± 0.65^b^	14.7 ± 1.14^a^

The averages shown with different letters (a–d) in the same line are significantly different at p < 0.05.

*MN* micronucleus, *FG* fragment, *VCR* vagrant chromosome, *SCR* sticky chromosome, *UDC* uneven distribution of chromatin, *BR* bridge, *NVC* nucleus with vacuoles, *RPL* reverse polarization, *IM* irregular mitosis.

The CAs test, along with the MN test, is among the most accomplished genetic bioassays with high sensitivity for early impacts^39,40^. The genotoxicity assessment in *A. cepa* roots indicated that TFS caused various CAs (Fig. 2, Table 2). Indeed, even the lowest dose of TFS solution applied was sufficient to induce CAs production in *A. cepa* root cells. The most observed CAs in all groups following TFS treatments was fragment (FR) (Fig. 2b, Table 2). FR was not found in the control group similar to MN. On the other hand, the differences between the FR incidences of the TFS-exposed groups were significant. There was a substantial enhancement in the frequency of vagrant chromosomes (VCRs) (Fig. 2c, Table 2) following TFS administrations. VCR was the second most observed CAs in all TFS-exposed groups and the increase in TFS-induced accumulation was dose-dependent. It is well-known that FR is an irreversible mutation that eventually results in the creation of MN^41^, while VCR accumulation causes the generation of dissimilar shaped- and sized-nuclei with different numbers of chromosomes in daughter cells^42^. Sticky chromosome (SCR) (Fig. 2d, Table 2), the third most seen CAs in *A. cepa* cells after treatment with TFS, is a chromosomal agglomeration that results from degeneration or depolymerization of DNA^43^. It is characterized as a cluster that is formed due to the improper folding of the chromosomal fibers into individual chromosomes or chromatid fibers^44^. Again, dose dependence was valid in the potential of TFS to induce sticky chromosomes (Table 2). Another CAs type accumulated following TFS treatments was uneven distribution of chromatin (UDC) (Fig. 2e, Table 2). According to Dutta et al.^45^, the UDC pattern is a consequence of the failure of chromatids to separate during anaphase and is an obvious factor for VCR generation. Normally dividing cells in the control group had no bridge (BR) (Fig. 2f, Table 2), nucleus with vacuoles (NVC) (Fig. 2g, Table 2), reverse polarization (RPL) (Fig. 2h, Table 2) or irregular mitosis (IM) (Fig. 2i, Table 2). On the other hand, similar to other types of CAs, the frequency of such aberrations also tended to increase with increasing TFS doses in the treatment groups. BR, a visible sign of clastogenicity or genotoxicity, occurs between chromosomes^42^ and leads to laggard chromosomes because of the formation of stickiness, unequal chromatid change, dicentric chromosomes or chromosomal breaks^45,46^. Malakahmad et al.^47^ noted that double-stranded ruptures in DNA cause the generation of chromosomal rearrangements or translocations, including stickiness or lagging. NVC is an abnormality that arises from the malfunctions in DNA biosynthesis during the synthesis stage of the mitotic cycle^34^, while RPL indicates defects in the organization of the spindles^48^. Youssef and Elamawi et al.^49^ stated that NVC reflects cytological damage and may be linked to the absence of genetic material in the nucleus. According to Rank et al.^50^, two possible mechanisms for the generation of CAs in mitotic plant cells depend on the action mechanism of the genotoxic material: aneugenic materials interrupt the formation of spindles or prevent the attachment of tubulins to kinetochores, while clastogenic materials trigger DNA ruptures. According to the results of our study, all doses of TFS induced both ways of CAs formation in *A. cepa* root cells. The genotoxic and cytotoxic effects of TFS in *A. cepa* have never been studied previously, however Wu et al.^9^ reported that TFS induces DNA damage in earthworms. In addition, Pérez et al.^40^ indicated that a different type of strobilurin, Azoxystrobin, triggers spindle failure-related CAs, including laggard chromosomes and failures in the congregation of chromosomes at the equator of the cell during metaphase in *Bidens laevis* L. It has also been reported that Azoxystrobin leads to DNA damage depending on the application dose of the fungicide in zebrafish liver^51^. TFS-induced increases in CAs and MN frequencies may be the primary causes of reduction in MI and plant growth in *A. cepa* because they decrease cell viability.

### Biochemical effects of TFS

Assessment of the biochemical parameters showed that TFS treatments induced striking oxidative stress in *A. cepa* root cells (Table 3). Oxidative stress is defined as disturbed redox equilibrium in the cells arising from the over-accumulation of reactive oxygen species and inadequate antioxidant defense^52^. Treatments with TFS at all doses triggered remarkable elevations in the catalytic activities of SOD and CAT. The mean SOD activities of the groups treated with 0.2 g/L TFS, 0.4 g/L TFS, and 0.8 g/L TFS were approximately 1.2, 1.5, and 2.0 times those of the control group, respectively. On the other hand, the mean CAT activities of the groups treated with 0.2 g/L TFS, 0.4 g/L TFS, and 0.8 g/L TFS were approximately 1.4, 1.8 and 2.4 times those of the control group, respectively. SOD and CAT enzymes work as a team in the enzymatic antioxidant system in cells. While SOD activity reduces superoxide radical and produces hydrogen peroxide and dioxygen, hydrogen peroxide is rapidly consumed by the catalytic activity of the CAT enzyme^53^. Mohsin et al.^54^ suggested that low doses of TFS help antioxidant defense in plant cells by improving the antioxidant activity of the enzymes. Similarly, Pyraclostrobin reduced oxidative stress by increasing SOD activity in barley^55^. Moreover, Azoxystrobin exhibited a protective potential in wheat through the enhancement of the activities of SOD, CAT and peroxidase enzymes^56^. In contrast, several studies on aquatic or terrestrial animals have shown that TFS induces oxidative damage^5^. In addition, Li et al.^57^ revealed that SOD and CAT activities were reduced in zebrafish embryos following treatments with strobilurins, including TFS. In our study, TFS obviously increased both the antioxidant enzyme activities and the MDA accumulation dose-dependently (Table 3). MDA is a low molecular weight product produced by the breakdown of membrane lipids during the attack of reactive oxidative species on the cellular membranes^58,59^. The mean MDA levels of the groups treated with 0.2 g/L TFS, 0.4 g/L TFS, and 0.8 g/L TFS were nearly 1.4, 2.1 and 2.7 times those of the control group, respectively. Mohsin et al.^60^ suggested that TFS reduces MDA levels by supporting antioxidant enzyme activities during salt stress in cucumber seedlings. On the contrary, TFS administration induced MDA accumulation in rare minnows due to the onset of oxidative stress^61^. Cellular microstructure studies on *Chlorella vulgaris* showed that TFS negatively affected membrane permeability and led to irreversible injuries on the cell membranes^62^. To our knowledge, our study is the first to demonstrate a TFS-related oxidative burst in *A. cepa* roots. It can be assumed that these results are related to the direct contact of the solutions containing high doses of TFS with the roots of *A. cepa*.

**Table 3:** Effect of Trifloxystrobin on selected biochemical parameters.

Groups	SOD (U/mg FW)	CAT (OD_240 nm_/min g FW)	MDA (µM/g FW)
Control	37.8 ± 3.98^d^	1.16 ± 0.54^d^	10.6 ± 1.14^d^
TFS 0.2 g/L	45.9 ± 4.76^c^	1.59 ± 0.69^c^	15.4 ± 1.41^c^
TFS 0.4 g/L	56.1 ± 5.82^b^	2.13 ± 0.90^b^	21.7 ± 1.70^b^
TFS 0.8 g/L	75.2 ± 6.84^a^	2.78 ± 1.16^a^	28.8 ± 2.13^a^

The averages shown with different letters (a–d) in the same column are significantly different at p < 0.05.

### Effects of TFS on meristematic cells

**Figure 3:**
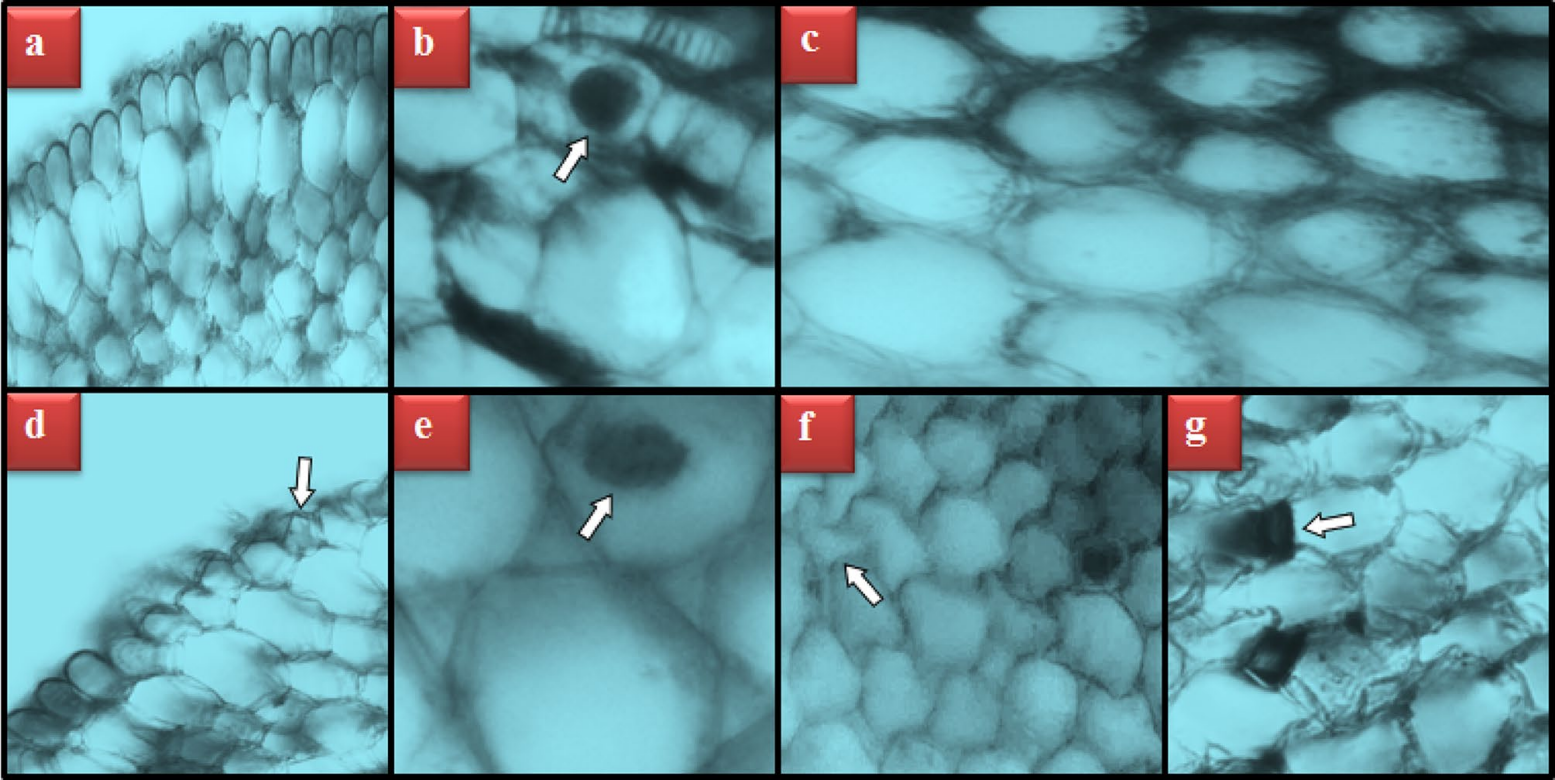
Meristematic cell damages induced by Trifoxystrobin. Appearance of healthy epidermis cells (**a**), appearance of healthy cell nucleus (oval) (**b**), appearance of healthy cortex cells (**c**), damaged epidermis cells (**d**), flattened cell nucleus (**e**), damaged cortex cells (**f**), thickness in cortex cell wall (**g**).

TFS treatments applied at all doses disturbed the integrity of *A. cepa* root meristematic tissue (Table 4, Fig. 3). Root meristem cells of the bulbs in the control group appeared healthy (Fig. 3a–c), while damaged epidermis (Fig. 3d), flattened cell nucleus (Fig. 3e), damaged cortex cells (Fig. 3f) and thickness in cortex cell wall (Fig. 3g) were detected in TFS-exposed root meristems. All abnormalities were at a slight level in the group treated with 0.2 g/L TFS. On the other hand, in the 0.4 g/L TFS-exposed group, epidermis damage and flattened cell nucleus scores ascended to a moderate level and damaged cortex cells and thickness in cortex cell wall remained at the same level. Meristematic cell damages were most severe in the TFS 0.8 g/L group. In this group, damage in the epidermis and flatness of cell nucleus reached critical points, while damages on cortex cells and the thickness in the cortex cell wall were moderate. Although strobilurins have been shown to promote root growth by acting like auxins at low doses in many plants^28^, TFS caused severe anatomical damage in *A. cepa* at high doses. Uçkun and Özmen^63^ stated that membrane permeability and osmoregulation were impaired due to possible cell membrane structural damage in *Xenopus laevis* tadpoles exposed to TFS. As can be seen from the MDA results, the most likely cause of meristematic cell damage in roots is the damage to cell membranes by TFS. In addition, an unusually shaped nucleus may be related to genotoxicity. Baker^64^ mentioned that the thickening of the cortex wall may be a defense mechanism of plants to prevent the transfer of harmful chemicals to other tissues. Meristematic cell damages such as epidermis deformation, abnormal cortex cell wall, necrosis, unclear vascular tissue and cortex cells with impurity due to fungicides have been previously demonstrated in *A. cepa* by Güç et al.^65^ and Demirtas et al.^66^.

**Table 4:** Degree of meristematic cell damages induced by Trifoxystrobin.

Groups	DE	FCN	DC	TCCW
Control	**−**	**−**	**−**	**−**
TFS 0.2 g/L	**+**	**+**	**+**	**+**
TFS 0.4 g/L	**++**	**++**	**+**	**+**
TFS 0.8 g/L	**+++**	**+++**	**++**	**++**

*DE* damaged epidermis, *FCN* flattened cell nucleus, *DC* damaged cortex, *TCCW* thickness in cortex cell wall, (−): undamaged, (+): slightly damaged, (++): moderately damaged, (+++): critically damaged.

### Conclusion

This study revealed the potential dangers of TFS in non-targeted biota by using *A. cepa*, as the model organism. TFS induced conspicuous failure in growth, genetic stability, cellular oxidative balance and meristematic tissue integrity. TFS-encouraged toxicity tended to increase as the doses of TFS increased. Despite the previous studies that mentioned the protective roles of TFS in plant stress tolerance along with the fungicidal effects, our data indicated that TFS should be considered genotoxic and cytotoxic at high doses. Therefore, the Paracelcus phenomenon "sola dosis facit venenum", which means “only the dosage makes the poison”, is valid for TFS.
